# Pelvic Hæmatocele

**Published:** 1886-04

**Authors:** W. W. Grant

**Affiliations:** 223 Brady Street; Davenport, Iowa


### REPORT OF A CASE OF PELVIC HÆMATOCELE.

*By* W. W. GRANT, M. D., *of Davenport, Iowa.*

Miss B., twenty-four years old, resident of Tennessee. A lady of education, culture and refinement, of delicate physical organization. On February 7th, 1881, on the third day of menstruation, while in the act of lifting a bed to take a trunk from under it, she was taken with a sudden, intense, and she said, an indescribable pain, of a tearing nature, in the region of the pelvis, and fell to the floor in a condition of syncope. She was taken up and put to bed, and continued to suffer from pain, extreme weakness, pallor and vomiting for several hours. Professor Madden was sent for and administered several hypodermic injections of morphine before relief was obtained. The menses stopped suddenly, and hot applications were applied "to bring back the flow," as it usually continued a week. But it did not return until the next regular period, when it was profuse. The attack was followed by much pelvic tenderness, slight tympanites and pains, sometimes of a spasmodic character, until July, 1881. With the consent of her physician she went, in less than two weeks from the time of attack, to Florida in company with her father for the benefit of her health. She suffered much *en route*, but after reaching her destination and keeping quiet she soon commenced to improve. The improvement in health continued until January, 1882, when pain, though not as severe as formerly, and swelling returned. Notwithstanding this fact, she went, on the 14th of February, to an evening party, during menstruation, participated in the dance and went home suffering again quite severely, and remained in bed for six months. During this time she was seen and treated by the well known surgeon, Professor Briggs. The case was regarded as one of pelvic cellulitis and peritonitis, with tendency to the formation of abscess, hæmorrhage never being suggested. The pain, tenderness and swelling diminished, but her general health gradually grew worse.

On 9th October, 1882, with the consent, if not advice, of her physician, Dr. Madden, she left Nashville on a second tour for the benefit of her health, her destination being Chicago and Davenport, where she had friends and relatives. The synopsis of her history was given to me by herself and family.

On October 28th, 1882, twenty months from the date of attack, I took charge of the case. The patient was suffering, though not greatly, from a feeling of discomfort, tension and heaviness in the hypogastrium, but most from a dull and unceasing pain in the lumbar and dorsal spine, which made her exceedingly restless, nervous and sleepless. From this condition she had at no time been entirely free. She rarely had a good night's rest. She was decidedly anæmic, and much emaciated. This condition with chilly sensations and slight febrile reaction occasionally in the evening suggested septicæmia. On examination I found a tumor which not only filled the pelvis on both sides but extended above the umbilicus, but with the patient on her back was most prominent in the right hypogastrium, in which locality she had suffered most pain from the beginning. On palpation the swelling was hard and unyielding. Even with the finger in vagina no fluctuation could be detected. The body of the uterus could not be defined, but the mouth and cervix rested on the floor of the perineum and was perfectly immovable, the os pointing up towards *ostium vaginæ.* To the finger in the vagina it was a hard, rather uniform swelling, the roof and surrounding parts as firm and unyielding as in severe peritonitis.

The beginning certainly indicated internal hæmorrhage. I determined to settle the nature and character of the case satisfactorily by aspiration, and if fluid was found, to evacuate it by this means at once. So I introduced the needle into the most prominent part of the swelling in the right hypogastrium. The appearance of a thick, tarry liquid—the first containing a good deal of pus—confirmed the belief of long-standing hæmatocele. With the patient in the recumbent position, only with head and shoulders slightly elevated by pillows, I continued the process of evacuation, only stopping to empty the bottle of the liquid, until, by accurate measurement, fifty-seven ounces had been withdrawn—the time being forty-five minutes. The patient now feeling faint I stopped the operation, hoping that the small quantity of the fluid left would be absorbed, and especially as the last seemed fresher and to contain no pus. The first pint of liquid contained much pus. The rest with the needle pushed to the depth of three inches seemed to contain none. The pain in the back, and every feeling of discomfort, was promptly relieved. She remained in bed two or three weeks, and improved steadily and rapidly. Menses appeared about the usual time of the month in November, and were more regular and natural than before. In December, contrary to instructions, the patient went to a party on a cold, stormy evening. The next day she commenced to suffer pain and a feeling of discomfort as before. An examination demonstrated that the sac was filling; sore on December 21st I again used the aspirator, removing the entire contents, twenty-six ounces of decomposed blood, and treated as before, with rest, iodide of potassium, tonics, and an abundance of milk with a good quantity of solid food. The patient progressed satisfactorily, the menses appeared at usual time; were quite free, but irregular, the flow stopping and reappearing for a week, but not attended with much pain. Her general health demanded out-door life, and she was given its advantages, as after the previous operation. She improved, but not as rapidly and uninterruptedly as I expected. She suffered from sleeplessness, slight pelvic pain, and slight feeling of weight in the pelvis. In January, 1883, menses appeared and pursued a course similar to the December period. In February, about the middle of the month, the exact date, by oversight, not being recorded, examination satisfied me that the fluid was again accumulating, so I again evacuated, as in the second operation, the entire contents, amounting by aspiration to sixteen ounces. There was no evidence of pus. Without removing the needle, the point of entrance being about the same each time, I injected, taking particular pains to exclude air, two ounces of the following mixture: compound solution iodine, one ounce, pure carbolic acid, one drachm, to four ounces of water, and allowed it to remain. The object being to obliterate the sac by inflammation and contraction. The injection induced, quickly, severe pain. The wall of the sac contracted energetically around the needle, as was shown by the fact that its withdrawal required some force. The pain was promptly relieved by a single hypodermic injection of morphia and atropia. A subacute inflammation followed, such as we would expect in a mild pelvic cellulitis or peritonitis. The temperature, never before above 99° F., was now 100° F. every evening for a week, and 99° F. in the morning.

The patient was not permitted to get up for any purpose. Required no further opiates except a suppository containing 1 grain aqueous extract of opium and ½ grain of the extract of belladonna at night for one week, for the purpose of inducing sleep, and to quiet and relieve the moderate pain. There was at no time difficulty in urination. A Spanish fly blister was now applied to the hypogastrium. Iodide of potassium and bichloride of mercury in combination were given three times a day for one month, and quinine also for two or three weeks. Hot water vaginal douches were freely administered for several weeks, and an abdominal binder quite firmly applied. The patient progressed satisfactorily, without a recurrence of the hæmorrhage, to a complete restoration of health. She remained in my charge still for several months, and then went to her home in Tennessee, well and happy. I have communicated with her repeatedly since, and she has, up to the present time, continued to enjoy most excellent health, horse-back riding being one means of exercise and pleasure.

This case presents several features of more than ordinary interest. The occurrence of this form of hæmorrhage in a young, unmarried lady; that with the best medical and surgical talent in Nashville error in diagnosis was evident; the large size of the tumor—being without a parallel so far as I can learn—and then too, the slight pain from it, except when first attacked, and in the back as stated, notwithstanding the constant increase in quantity of effused blood and the pressure and distention induced by it. The latter was so great that rupture of the sac must have been imminent. In spite of this state of things, this was a walking patient up to the very day of examination and use of the aspirator. I am not aware of another case being treated by aspiration and medicated injection in this way. Bearing in mind its long standing, that it was changing into an abscess, the immense size of the sac, the slight disturbance produced by the injection, and the steady and complete recovery, commends the plan of treatment worthy of a more extensive trial.

In the early stage hæmatocele does not demand operative interference. When the clot is not reabsorbed but liquefies, then its evacuation is required. There is a difference of opinion as to the best method of procedure. I believe aspiration deserves the preference, and especially in large effusions, because safer. It is more thoroughly antiseptic than any operation by incision can be. It does not expose a large surface to the action of the atmosphere, and suppuration is not likely to be established by it. When the contents are offensive or pus has formed, I should in the future, after evacuating the contents by aspiration, wash out the sac with warm water, and then inject iodine or carbolic acid, or both, and permit a certain quantity to remain, as in the case reported. I believe this was a case of extraperitoneal hæmatocele, commencing by a rupture of a vein beneath the right ovary, within the folds of the broad ligament, and that the hæmorrhage recurred with subsequent menstrual periods, dissecting the broad ligament and passing in front of the uterus, and to the left side.

223 Brady Street.

